# Reflex Pain

**Published:** 1884-10

**Authors:** Kate Camerom Moody

**Affiliations:** Mendota, Ill.


### THE DENTAL REGISTER.

VOL. XXXVIII.] OCTOBER, 1884. [No. 10.

### COMMUNICATIONS.

### Reflex Pain.

BY KATE CAMEROM MOODY, D.D.S., MENDOTA, ILL.

Read before the Illinois State Dental Society.

After having nearly completed this paper I learned that Dr. Newkirk had presented something in the same line of thought a few years ago before this Society; but, as it is a subject of a great deal of importance to us all, and upon which very little has been said, it seemed that a change of topic was not best.

When first entering upon active duties in my profession this was one of the most formidable mysteries I had to encounter; the complex nature of which was only equaled by my disappointment at finding so little, in all our literature, to give me light.

The common use of the word "reflex," as we all know, is the name given to that influence which, when exerted upon the periphery, or terminal branches of a nerve, is conveyed to the center by an afferent, or sensory fibre, to be "reflected" by an afferent or motor fibre; the reflex impulse being one of *motion*, and not of *sensation*. An example: We take hold of a hot iron; the impulse conveyed to the center gives a sensation of pain; the center immediately sends out an impulse through the motor fibres which causes the muscles to contract, and we drop the iron. This is "reflex" action, pure and simple. But there is another application of the word which is quite commonly made, and which is employed here, namely, the designation of perverted nervous function, which occurs simultaneously with phenomena produced by other than peripheral irritation—the difference being that the reflected impulse is one of sensation instead of motion, also called sometimes *sympathetic* pain; though it has been doubted by good authority whether the sympathetic system is capable of transmitting such influences.

There are three great centers of reflex action—the brain and cord, the stomach and digestive apparatus, and the reproductive system. When any one of these centers is disturbed the influence is likely to radiate in any and all directions. In this way disease may arise in portions of the body quite distant from the true seat of irritation; hence the difficulty in diagnosis when judging from the locality of the symptoms. If there be over-excitement or worry of the mental faculties, it does not necessarily follow that the *head* will be the first to cry out in pain; there may be disturbance of the *stomach* instead, or, if the digestive organs become disarranged, the head may be the first to give notice. Thus it is that, in attempting to cure disease by removing the cause, we sometimes find ourselves in deep water.

We have always been taught to define pain as "an impingement upon a nerve." Let us consider it as in abstract. Physiologically, what is it? or, shall we say *pathologically?* for there can be no such thing, of course, as physiological pain. What is that condition of the nervous system which our consciousness interprets as "pain." Is it, as some claim, only an excess of ordinary sensory functions? The function of any organ or tissue is the work it does when in a healthy condition. The work of the nerve is to convey impulses to and from the center; then, if some other than normal work is done by a nerve, we conclude that something is wrong, some change *somewhere* causing this perverted function.

It is within the delicate mysterious chambers of the brain where resides this hard-to-be defined influence or sensation we call "pain." It is here the change from afferent to efferent impulses takes place. When, therefore, pain is felt in a part, as the result of reflex influence of some remote diseased part, may not the fault lie in the *center*, which fails to transmit correctly? thus making the secret of reflex pain a *psychological* and not a *pathological* one. How, otherwise, explain the instantaneous relief from reflected pain on the removal of the real cause? Can there be real molecular changes existing which would cease immediately, without time for the usual repair of tissue necessary to complete absence of pain? Tuke, in his "Influence of the Mind upon the Body," says: "Emotional impulses may act upon the sensory ganglia and nuclei of the nerves of sensation so as to produce any of those sensations which are ordinarily induced by impressions upon their periphery; such sensations, although central, being referred by the mind to the peripheral terminations of the nerves."

To return to the work performed by the nerves. In what does this change of function consist? The cutaneous nerves convey to the center a sense of comfort when the surrounding atmosphere is neither too hot nor too cold; but let these same nerves be exposed to a cold wind, and quite a different sensation is produced, which causes the reflex movements of shivering, or, if long continued, of aching pain. In the same way intense heat, when applied to the surface, will cause pain. Now, both heat and cold produce an agreeable sensation when applied in a certain degree to the surface, but an entirely different sensation when the degree is increased. So we see that if a force which produces an agreeable sensation be increased to a certain extent, the result is a sensation of pain. Then does it not seem that the difference between comfort and pain is one of intensity only, *quickened impulses*, just as the difference in wave lengths of light will produce different colors; or may not the different results produced by nervous impulses be due merely to a difference in wave lengths of the impulse—or, in other words, only a "mode of motion?" A nerve, like a faithful messenger, is going about its daily duties, performing its normal functions—an impingement occurs—*it merely hurries up to tell the news!*

It is not necessary that any tissue of the body should be putting forth its greatest effort when producing normal results. Nature has provided, in various way, against accident and disease in our bodies. A blood vessel is not distended to its utmost when simply carrying the blood at a normal rate; the large sinuses of the brain provide against an engorgement or obstruction in that important part; the heart possesses a capacity which permits great acceleration of motion in case of excitement; the lungs, likewise. A man may walk three miles in a leisurely way and feel refreshed, but if he put forth extra effort, and walk five mile in the same length of time, he will become fatigued, although he use the same muscles for the work. But what of the nature of this force? Who shall say? Will scientists finally solve the question?

In a recent scientific journal the similarity between nerve force and electrical force is discussed, and their identity well nigh proven, or, at least, nearly enough so to make the theory seem a very plausible one.* This, in connection with the late experiments of Prof. Hughes, of England, by which he has shown all matter to possess an inherent property, manifested to us, under certain conditions, in the phenomena of magnetism and electricity makes a strong argument in assuming this nerve force to be but another manifestation of this same mysterious property common to all matter.

It is through the vast net work of nervous structure, interwoven, crossing, recrossing, and uniting, like lines of telegraph, in every part of the body, that these impulses are constantly being conveyed; their connections and relations being so intimate that one portion of the body cannot suffer alone but a deep sympathy is found to exist in the surrounding parts, and if the injury be great the whole body is involved. Whether these impulses are conveyed entirely by the sympathetic system of nerves, or whether through the continuity of fibres at the origin of the main nerves is a question. It is a fact that the fibres of some of the main nerves may be traced to the same point in the medulla, either in their deep or superficial origin. Anstie speaks of the close juxtaposition and intimate reflex relation existing between the roots of the fifth cranial and pneumogastric, so that the impressions made upon the fifth, in extracting, exert a powerful stimulating effect upon the pneumogastric. Why may not the pain in the stomach and œsophagus, "the lump in the throat" of hysterical patients, after an operation be due to the same cause? Irritation of the fifth has been known to cause violent fits of

*Scientific American, May 3d, 1884.

vomiting and cardiac pains, as in the eruption of the third molar.

Of all nerves in the body the fifth cranial is the most interesting to us, as dentists, not merely because its branches supply the dental organs, but because it is most often the seat of neuralgic affections, with the exception perhaps of the sciatic, and when thus affected, yields less readily to treatment than any other nerve. Also, owing to its large and much exposed peripheral expanse, the complex nature of its functions and its close connection with other important nerves, its affections are most likely to cause secondary or "reflex" disturbance of wide extent. Without taking this into consideration, it would seem strange that cervico-brachial neuralgia, or disturbance of digestion, should be caused by carious teeth. Yet not only these, but parts more distant will often become thus affected. There seems to be an especially close relation existing between the teeth and the reproductive organs, and, at times, when the latter are peculiarly engaged, it is not an uncommon occurrence for us to be called upon to treat perfectly sound teeth, though of course more often unsound ones. Again the careless, or shall we say *ignorant*, practitioner, will sometimes remove a sound tooth without relief to the patient, when the true cause of the trouble may lie no further away than the opposite side of the mouth. Simply because a person, almost insane with pain, demands treatment of a certain tooth, is no reason why a cool-headed dentist should not make a thorough examination of not only all the teeth, but, by using his knowledge of nervous distribution, inquire into the general systemic condition. We do not wish to be understood as asserting that such an examination and inquiry will invariably bring to light the lurking cause, but that the removal of an obscure cause does very frequently produce the desired result, is sufficient reason for the exercising of more care, yes, *more knowledge* than is sometimes used.

I am aware that the temptation is sometimes strong to use our skill only so far as we are compensated therefor; and yet, is it the highest aim of our profession to accumulate wealth? Is there not a more noble object to be gained, namely, the alleviation of suffering, as well as the more selfish one of self-improvement, in adding to our knowledge of the delicate structures under our care? We cannot be too well informed on these things; besides, it behooves us, as specialists, to not only post ourselves on what others have found out by experiment and investigation, but also to experiment and investigate for ourselves. Let us not leave it to the medical profession to prepare our food for us, being content with opening our mouths to swallow the bolus when ready.

An eminent medical authority, in his review of the state of ophthalmology for the year 1883, says: "The influence of carious teeth in producing affections of the eye is neither sufficiently considered nor understood. The relation between the two is often very noticeable."* Do not we, as dentists, share this implied reproach upon the medical profession? We are willing enough to confess our ignorance; but this is not sufficient. Let us "*observe, compare, reflect, record.*" There is ample room for research upon this one subject of "reflex pain." As I said before, the meagerness of our literature upon this subject, to one in search of facts, is very striking. It cannot be that there are not frequently occurring, in our many dental offices, such incidents, which, if recorded, with the results of proper research, would help many another, who may be puzzling over the strange occurrences which baffle all his skill. Let us honestly and faithfully record the results of our research—not being too fearful of criticism—and thus help to diffuse the knowlege we all need. If you have a patient with an inflamed and painful eye, which has refused to be comforted by all other treatment, but is immediately relieved after the treatment of a carious tooth, don't keep the good news to yourself, but "call your neighbors in," and divide; after first satisfying yourself as to the probable reason of this sympathetic action between the different branches of the nerve, whether it be due to want of tone of the system, or to whatever exciting or predisposing cause, make a short and concise note of it, and send it to one of our journals, which will gladly assist you in your efforts to do good by publishing it.

---

*Report on Ophthalmology. S. J. Jones, A.M., M.D., Chicago, 1883.

